# Supplementary material for: Systematic discovery and functional interrogation of SARS-CoV-2 viral RNA-host protein interactions during infection
Source: bioRxiv. 2020 Oct 6:2020.10.06.327445. Preprint. [Version 1] doi: 10.1101/2020.10.06.327445 (PMC7553159; doi:10.1101/2020.10.06.327445)

bioRxiv preprint doi: https://doi.org/10.1101/2020.10.06.327445. this version posted October 6, 2020. The copyright holder for this preprint (which was not certified by peer review) is the author/funder. It is made available under a CC-BY 4.0 International license.

Supplementary Figure Legends

Figure S1, related to Figure 1
(A) Host and viral RNA-seq alignment statistics for all samples across Huh7.5 (left) and VeroE6 (right) cell lines.

Figure S2, related to Figure 2
(A) High confidence SARS-CoV-2 human interactome network colored by time point (24 h.p.i., 48 h.p.i., or both). **(B)** High confidence SARS-CoV-2 human interactome network colored by species conservation.

Figure S3, related to Figure 2
(A) Comparison of the high confidence SARS-CoV-2 RNA associated human proteome by RAP-MS (UV crosslinking) to that by formaldehyde crosslinking (ChIRP-MS) and comparison of the SARS-CoV-2 RNA associated proteome to the SARS-CoV-2 protein associated proteome (PPI). **(B)** Overlap of interactomes by RAP-MS and ChIRP-MS. **(C)** Overlap of interactomes by PPI and ChIRP-MS.

Figure S4, related to Figure 4
(A) Pan-viral comparison of associations with translation initiation (EIF) factors.

Figure S5, related to Figure 5
(A) CRISPR guide residuals for SWI/SNF related genes enriched in the ChIRP-MS dataset. **(B)** All genes which are significant hits in the CRISPR screen (fdr ≤ 0.05) and also present in the high confidence interactome (smaller segment) or the expanded interactome (larger segment) of SARS-CoV-2 and their association with other viruses. Scale is capped at 5.

Figure S6, related to Figure 6
(A) Conservation between time points of SARS-associated host RNAs in the VeroE6 cell line. **(B)** Conservation between time points of SARS-associated host RNAs in Huh7.5 cell line. **(C)** ChIRP-MS enrichment of rRNA 2'-O-ribose methyltransferases across viruses.

Table Descriptions

Table S1: Sequences of 108 biotinylated ChIRP probes.

Table S2: Mass spectrometry data for viral proteins and host proteins with mean enrichment ≥ 1 in SARS-CoV-2 ChIRP-MS datasets.

bioRxiv preprint doi: https://doi.org/10.1101/2020.10.06.327445. this version posted October 6, 2020. The copyright holder for this preprint (which was not certified by peer review) is the author/funder. It is made available under a CC-BY 4.0 International license.

Table S3: Full data for all proteins in all datasets including pan-virus data, high confidence interactomes, expanded interactomes, CRISPR screen results, and presence in other datasets (such as UV-crosslinking data, PPI data).

bioRxiv preprint doi: https://doi.org/10.1101/2020.10.06.327445. this version posted October 6, 2020. The copyright holder for this preprint (which was not certified by peer review) is the author/funder. It is made available under a CC-BY 4.0 International license.

Figure S1

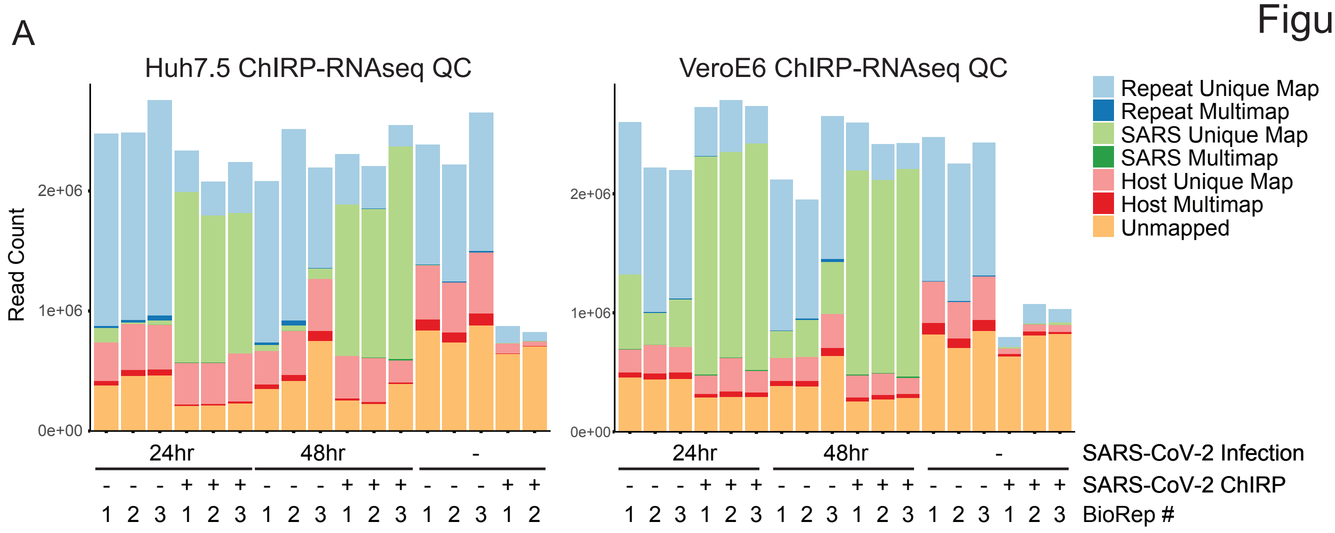

bioRxiv preprint doi: https://doi.org/10.1101/2020.10.06.327445. this version posted October 6, 2020. The copyright holder for this preprint (which was not certified by peer review) is the author/funder. It is made available under a CC-BY 4.0 International license.

Figure S2

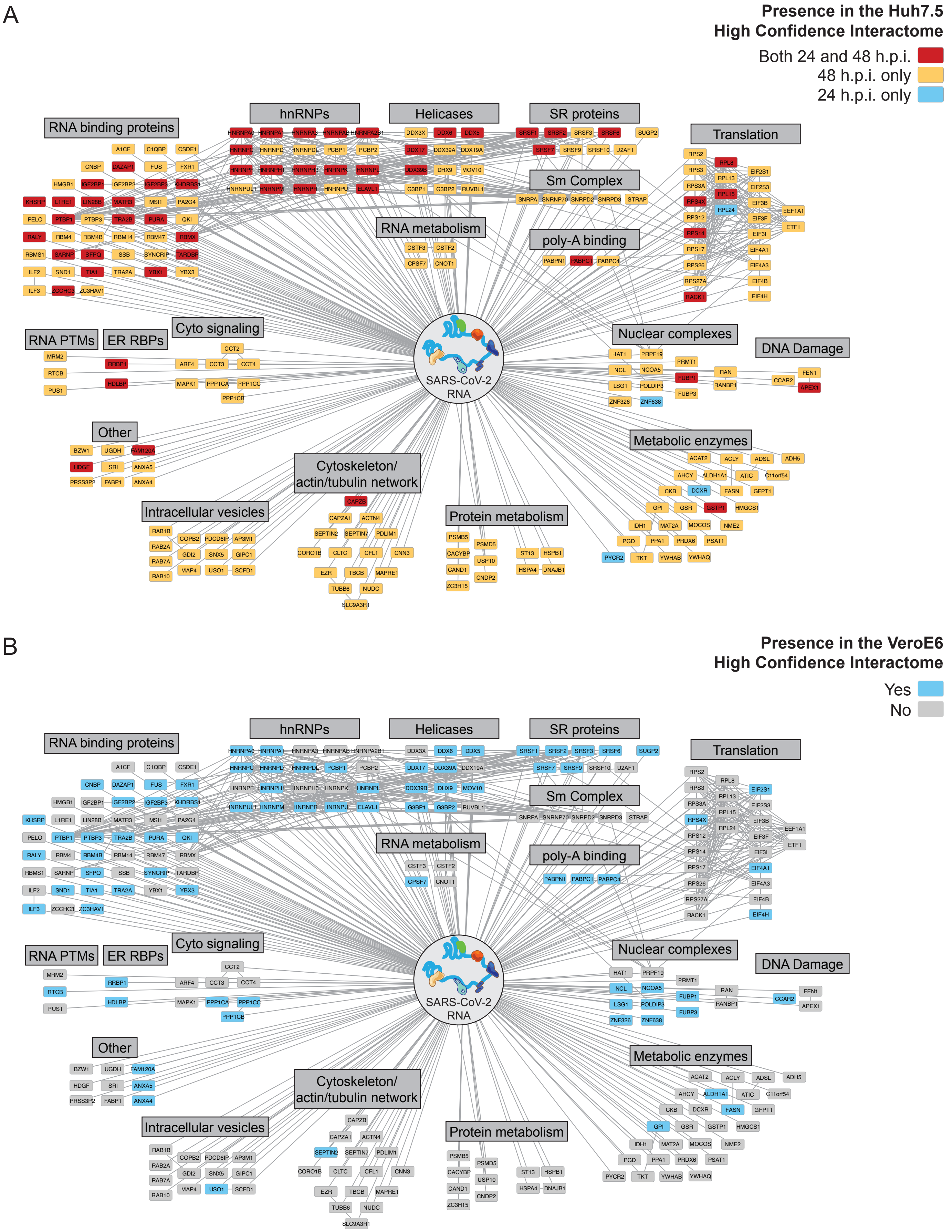

bioRxiv preprint doi: https://doi.org/10.1101/2020.10.06.327445. this version posted October 6, 2020. The copyright holder for this preprint (which was not certified by peer review) is the author/funder. It is made available under a CC-BY 4.0 International license.

Figure S3

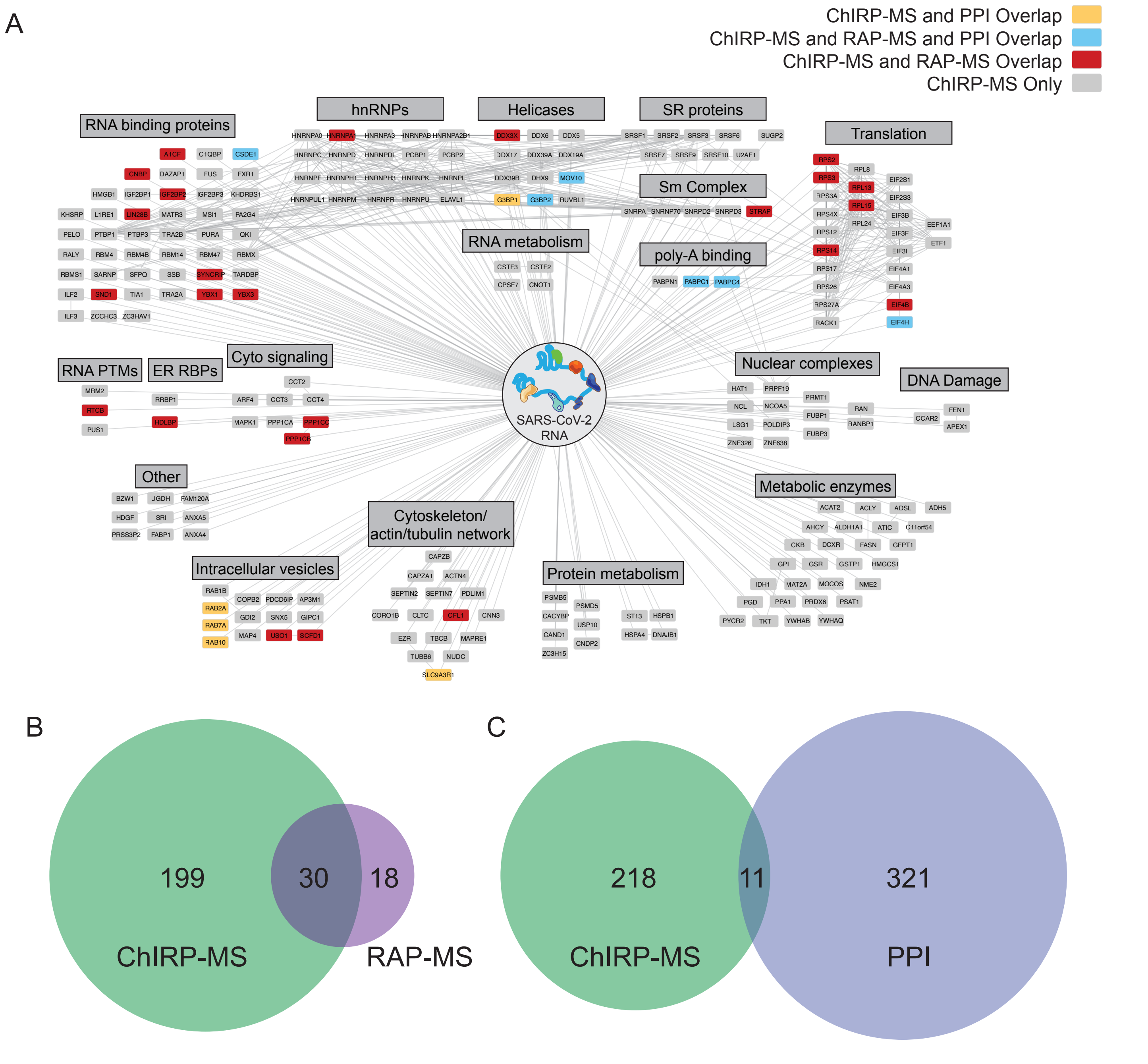

bioRxiv preprint doi: https://doi.org/10.1101/2020.10.06.327445. this version posted October 6, 2020. The copyright holder for this preprint (which was not certified by peer review) is the author/funder. It is made available under a CC-BY 4.0 International license.

Figure S4

A

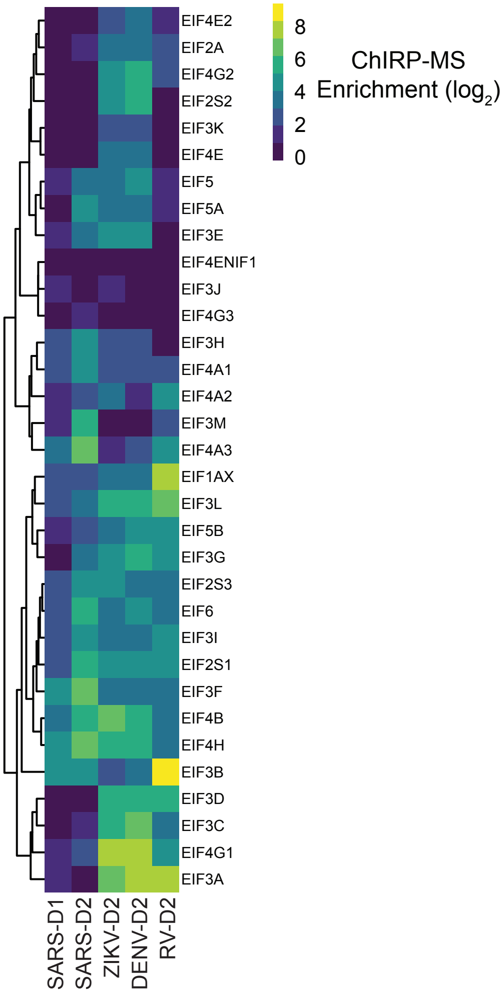

bioRxiv preprint doi: https://doi.org/10.1101/2020.10.06.327445. this version posted October 6, 2020. The copyright holder for this preprint (which was not certified by peer review) is the author/funder. It is made available under a CC-BY 4.0 International license.

Figure S5

A

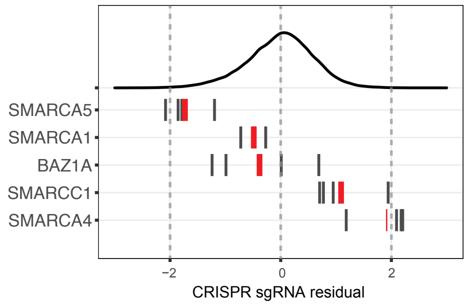

B

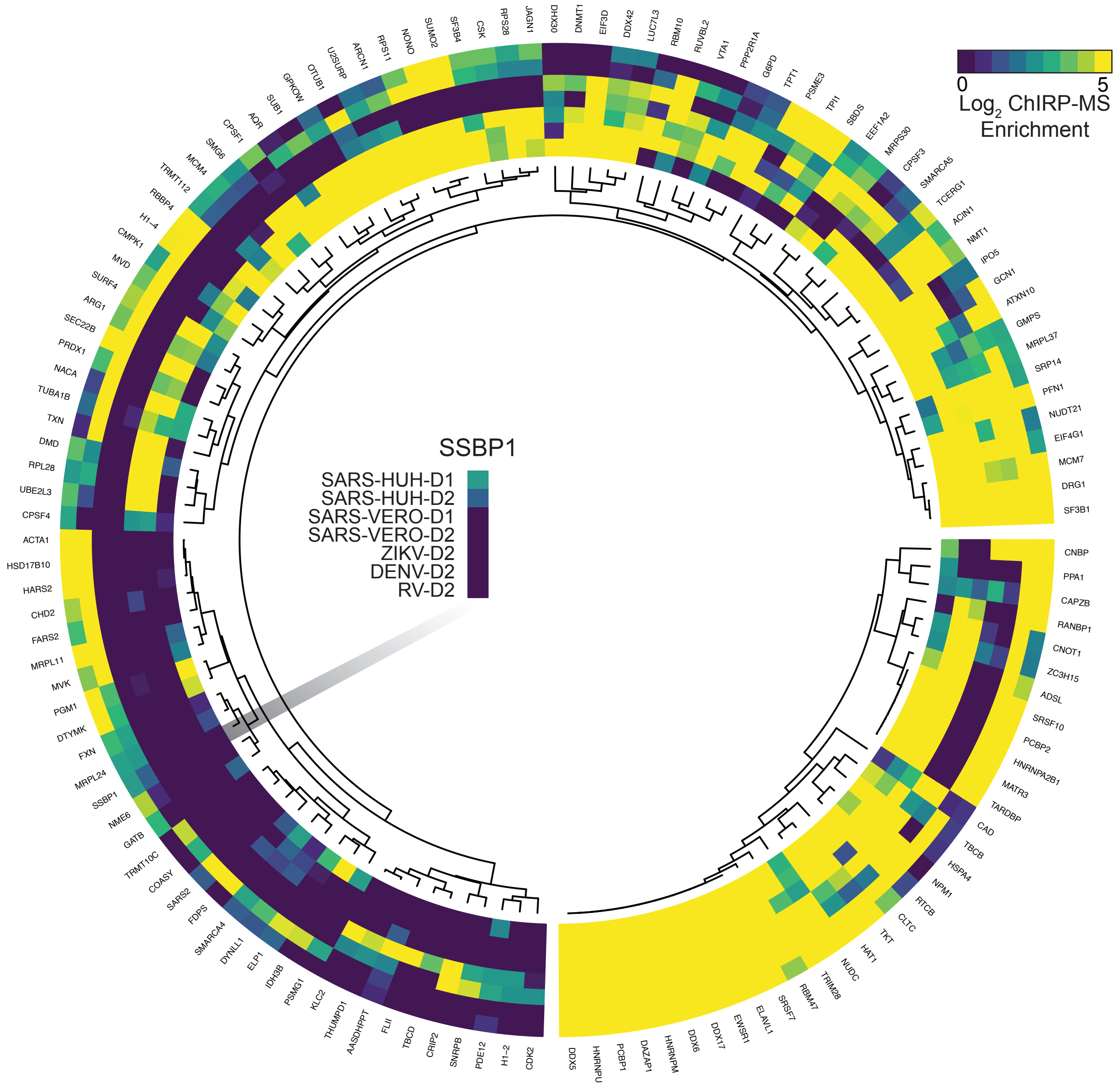

bioRxiv preprint doi: https://doi.org/10.1101/2020.10.06.327445. this version posted October 6, 2020. The copyright holder for this preprint (which was not certified by peer review) is the author/funder. It is made available under a CC-BY 4.0 International license.

Figure S6

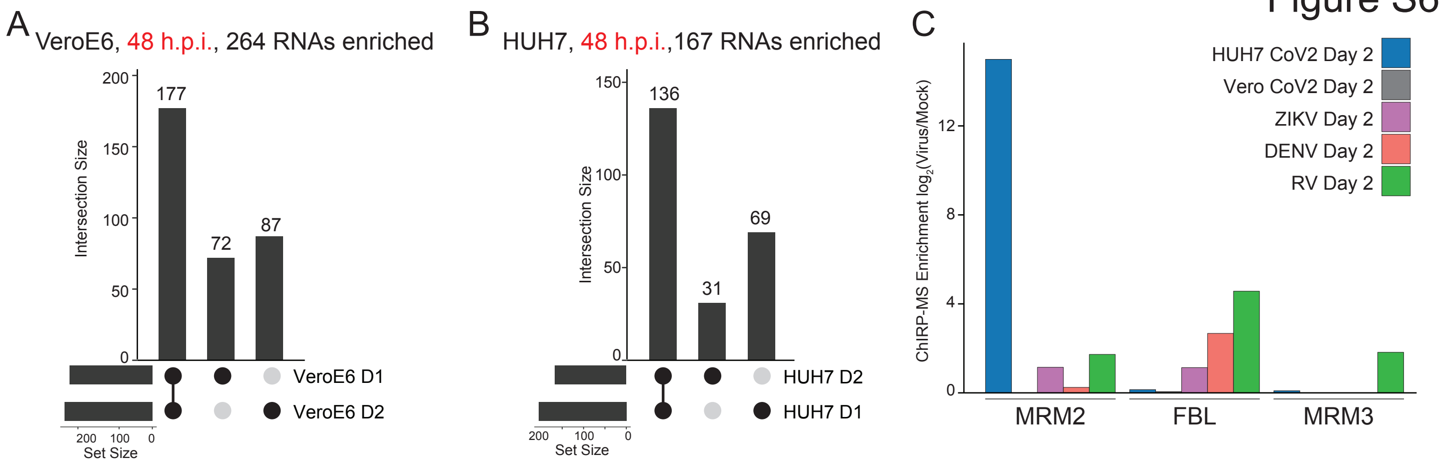

Supplement: 1 [file NIHPP2020.10.06.327445-supplement-1.pdf]
